# Intraoperative Platelet-Rich Plasma (PRP) for Post-Cesarean Scar Healing: A Single-Center Randomized Controlled Pilot Study

**DOI:** 10.3390/healthcare13222928

**Published:** 2025-11-15

**Authors:** Ana-Maria Brezeanu, Dragoș Brezeanu, Vlad-Iustin Tica

**Affiliations:** 1 6th Department, Faculty of Medicine, Ovidius University of Constanta, 900527 Constanta, Romania; anmariataras@gmail.com (A.-M.B.); vtica@eeirh.org (V.-I.T.); 2 County Clinical Emergency Hospital “Sf. Ap. Andrei”, 900500 Constanta, Romania

**Keywords:** platelet-rich plasma, cesarean section, scar healing, randomized controlled trial

## Abstract

**Background:** Cesarean section (CS) frequently results in abdominal scarring, affecting recovery, aesthetics, and quality of life. Platelet-rich plasma (PRP), an autologous concentrate rich in growth factors, may enhance wound healing. This pilot trial assessed the effect of intraoperative PRP on CS scar outcomes. **Methods:** In this single-center, single-blind randomized controlled trial (February 2023–December 2024), 100 women undergoing elective CS were randomized to PRP treatment (*n* = 50) or standard care (*n* = 50). PRP, prepared from 20 mL autologous blood, was infiltrated into uterine incision margins and subcutaneously before skin closure. Scar healing was evaluated at day 7 and day 40 postpartum using the Patient and Observer Scar Assessment Scale (POSAS; physician and patient), Vancouver Scar Scale, Manchester Scar Scale, REEDA (Redness, Edema, Ecchymosis, Discharge, Approximation) Scale, Visual Analog Scale (VAS), and Numeric Rating Scale (NRS). Mann–Whitney U tests and Cohen’s d effect sizes were calculated. **Results:** Follow-up was complete for all participants. On day 7, PRP-treated patients had lower mean scores across most scales (e.g., Vancouver: 1.74 ± 1.58 vs. 2.54 ± 2.30; *p* = 0.063). At day 40, improvements persisted, with POSAS Patient scores significantly lower in the PRP group (7.24 ± 1.81 vs. 8.00 ± 2.06; *p* = 0.029). Effect sizes were small-to-moderate (<0.5), suggesting underpowering. No adverse events occurred. **Conclusions:** PRP administration during CS showed favorable trends toward improved scar quality and reduced patient-reported discomfort, with statistical significance for POSAS Patient scores at 40 days. Larger, multicenter trials with extended follow-up are needed to confirm these findings.

## 1. Introduction

Cesarean section (CS) remains one of the most frequently performed surgical procedures in obstetrics, representing a vital intervention for the management of various maternal and fetal complications. However, it is invariably associated with the formation of an abdominal scar, which may significantly impact both the patient’s physical recovery and psychological well-being [1]. Since 1985, the World Health Organization has advocated for an optimal cesarean delivery rate of 10–15%, based on epidemiological and clinical outcome data [2]. Despite this recommendation, cesarean section rates have shown a steady increase worldwide, with recent estimates indicating global prevalence rates of 15.9% in Asia, 19% in Europe, and only 3.5% in Africa [3,4].

Scar formation is an inherent component of the wound healing process—a highly coordinated cascade involving inflammation, proliferation, and tissue remodeling [5]. In the context of cesarean delivery, postoperative scarring raises both aesthetic and functional concerns, with a direct impact on quality of life and future surgical outcomes [6,7]. Consequently, enhancing the healing process of surgical wounds and minimizing scar formation remain critical goals in postoperative care.

Platelet-rich plasma (PRP), an autologous biological product obtained by centrifugation of the patient’s own blood, has garnered significant attention in regenerative medicine due to its high content of growth factors—such as platelet-derived growth factor (PDGF), transforming growth factor-beta (TGF-β), and vascular endothelial growth factor (VEGF)—which are known to modulate inflammation, stimulate angiogenesis, and promote tissue repair [8,9]. Since its initial use in cardiac surgery in the late 1980s [10], PRP has been employed across multiple disciplines, including dermatology, orthopedics, and plastic surgery, with promising results in improving wound healing and reducing scar formation.

The present study aims to assess the efficacy of intraoperatively administered PRP during cesarean section on postoperative scar healing. By utilizing six validated scar evaluation scales at both 7 and 40 days post-surgery, we seek to determine whether PRP can significantly accelerate the healing process and enhance the overall quality of cesarean section scars. While prior pilot studies have examined the role of PRP in post-cesarean wound healing, our study introduces several novel elements. PRP was administered at two anatomical levels (uterine incision and subcutaneous tissue), allowing for a dual-phase regenerative effect assessment. Moreover, we utilized six complementary clinical and patient-reported scales to provide a multidimensional evaluation of scar outcomes. Finally, this study incorporated post hoc power and sample size calculations to inform the design of future definitive trials—an approach not previously reported in comparable obstetric studies.

Despite these promising findings, PRP therapy is not without limitations. Reported drawbacks include the absence of standardized preparation protocols, variability in platelet concentration and bioactive content, and the need for specialized centrifugation equipment. Moreover, PRP efficacy can be influenced by patient-related factors such as platelet count, hematocrit, and comorbidities. Although adverse events are rare, possible risks include local infection, transient pain, swelling, or minor bruising at the injection site. Acknowledging these challenges is essential to place PRP use in a realistic clinical context and to guide optimization in future research.

## 2. Materials and Methods

### 2.1. Study Design and Registration

This was a prospective, single-center, randomized controlled pilot trial evaluating the effect of intraoperative platelet-rich plasma (PRP) on postoperative cesarean section (CS) scar healing. The study was conducted at the County Clinical Emergency Hospital “Sf. Ap. Andrei”, Constanța, Romania, between February 2023 and December 2024, in accordance with the Declaration of Helsinki and Good Clinical Practice (ICH-GCP) guidelines. Ethical approval was obtained from the Ethics Committees of both Ovidius University of Constanța and the County Clinical Emergency Hospital “Sf. Ap. Andrei” (approval no. 7230/31.01.2023). Prior to participant enrollment, a procedural clarification restricting inclusion to elective cesarean sections (for protocol consistency and to minimize confounding by surgical urgency) was reviewed and accepted by the ethics committees as part of the approved protocol.

The trial was prospectively registered at ClinicalTrials.gov (Identifier: NCT06978010). The registry entry specified 100 participants randomized 1:1 to PRP treatment or standard care, with primary outcomes assessing postoperative scar quality (Patient and Observer Scar Assessment Scale—POSAS) at day 7 and day 40, and secondary outcomes including Visual Analog Scale (VAS) and REEDA scores.

### 2.2. Participants (Eligibility and Recruitment)

Women aged 18–42 years scheduled for elective cesarean section under spinal anesthesia were eligible. Exclusion criteria comprised body mass index > 35 kg/m^2^, keloid or hypertrophic scar history, diabetes mellitus, connective-tissue or hematological disorders, anticoagulant therapy, or inability to attend both follow-up visits. Recruitment occurred consecutively in the operating theatre after written informed consent. Although the initial trial registration permitted both elective and emergency cesarean sections, before participant enrollment commenced, the study team submitted a procedural clarification to the institutional ethics committee specifying that only elective cesarean sections would be included. The ethics committee confirmed that this refinement did not alter the risk–benefit profile of the study and approved the restricted inclusion criterion as part of the validated study protocol.

### 2.3. Randomization and Allocation Concealment

**Figure 1:**
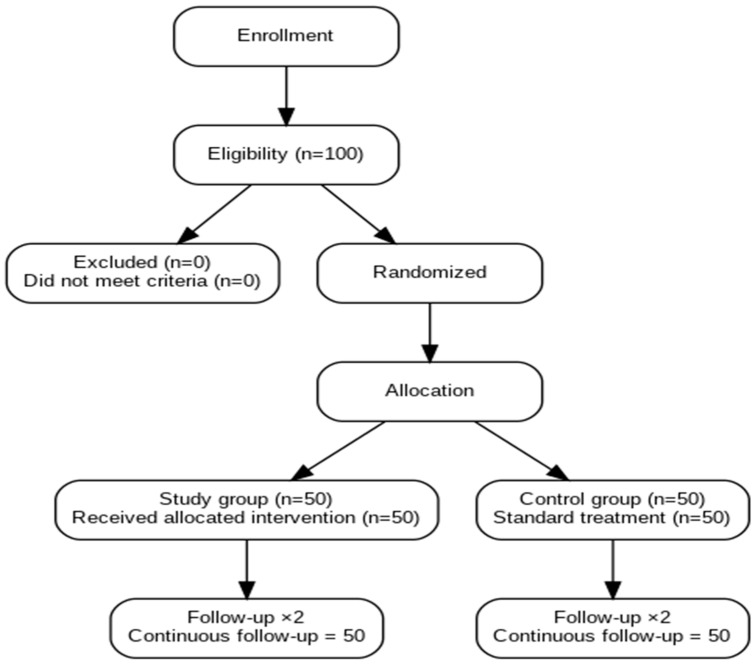
Consort Flow Diagram.

Participants were randomly assigned (1:1) using a computer-generated randomization sequence (randomization.org) as shown in Figure 1 and Table 1. Sealed opaque envelopes ensured allocation concealment until intraoperative assignment. Surgeons were aware of group allocation; however, postoperative data analysis was conducted by a statistician blinded to treatment assignment.

### 2.4. Intervention and Control Protocols

**Figure 2:**
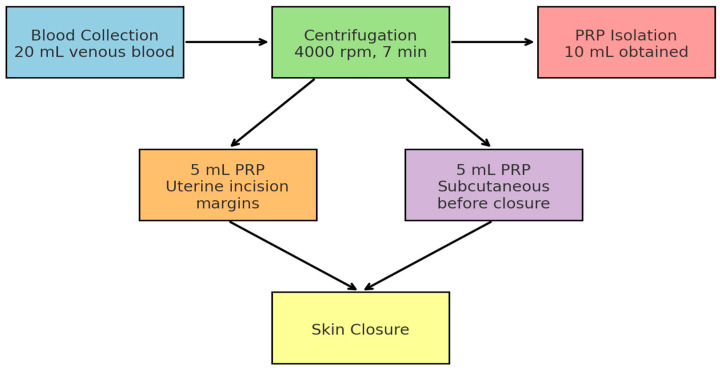
Workflow of Intraoperative PRP Application during Cesarean Section. The diagram illustrates the sequential steps of PRP use: venous blood collection (20 mL), centrifugation at 4000 rpm for 7 min, isolation of 10 mL PRP, followed by 5 mL infiltration into uterine incision margins and 5 mL subcutaneously before skin closure.

**Figure 3:**
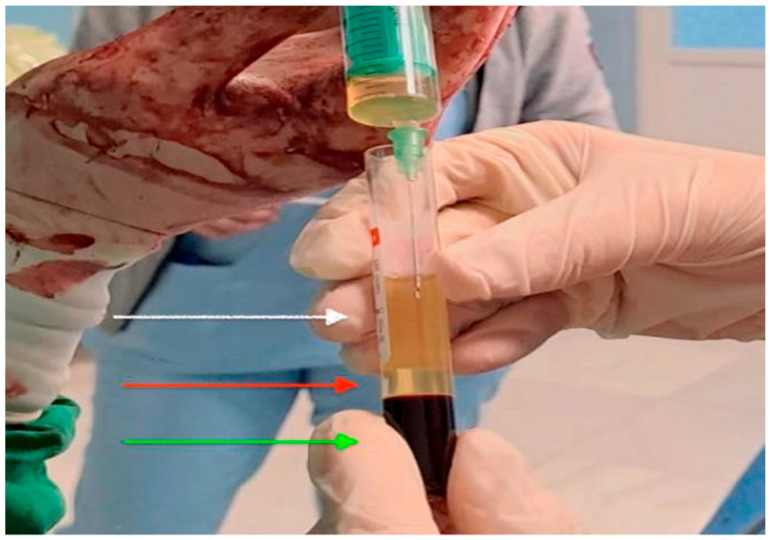
Intraoperative image of platelet-rich plasma prepared for application. The upper layer of platelet-poor plasma (white arrow), the middle layer of platelet-rich plasma (red arrow) and the bottom layer is the red blood cells (green arrow).

In the intervention arm, 20 mL of autologous venous blood was collected immediately after spinal anesthesia and centrifuged at 4000 rpm for 7 min using an XC Spin Plus centrifuge (XCmed, Bucharest, Romania). Approximately 10 mL of PRP was obtained as shown in Figure 2 and Figure 3.

Step 1: 5 mL infiltrated along the uterine incision margins before hysterorrhaphy.

Step 2: 5 mL injected subcutaneously before skin closure.

The control arm received standard cesarean closure without PRP. All other intraoperative and postoperative procedures followed identical protocols.

### 2.5. Outcome Measures

Scar healing was evaluated at day 7 and day 40 postpartum using six validated clinical and patient-reported instruments as shown in Table 2. Each scale was selected for its established reliability and reproducibility in surgical and obstetric wound healing research:

Patient and Observer Scar Assessment Scale (POSAS): This scale consists of two complementary components. The observer scale evaluates vascularity, pigmentation, thickness, relief, pliability, and surface area, while the patient scale assesses pain, itching, color, stiffness, thickness, and irregularity. POSAS provides both objective and subjective perspectives on scar quality, making it highly suitable for postpartum follow-up.

Vancouver Scar Scale (VSS): One of the most widely used tools for postoperative and burn scars, assessing vascularity, pigmentation, pliability, and scar height. It allows objective comparison between groups in clinical trials.

Manchester Scar Scale (MSS): This scale evaluates color, contour, texture, and overall distortion. It is known for its good inter-observer reliability and clinical utility in obstetric surgery scars.

REEDA Scale (Redness, Edema, Ecchymosis, Discharge, Approximation): Originally developed for perineal wound assessment, REEDA has been validated as a practical, sensitive tool for early surgical wound healing evaluation, particularly during the inflammatory phase.

Visual Analog Scale (VAS): A 10 cm horizontal line ranging from “no pain” to “worst imaginable pain,” completed by the patient to quantify subjective scar-related pain intensity.

Numeric Rating Scale (NRS): A patient-reported measure in which pain intensity is scored from 0 (no pain) to 10 (worst pain). It complements VAS by providing a quick, reproducible measure of discomfort.

In all assessment tools, lower scores reflect superior wound healing, better scar quality, and decreased patient-reported discomfort.

The combination of these scales allowed for a comprehensive evaluation of scar outcomes, capturing both clinical dimensions (vascularity, thickness, contour, inflammation) and patient-centered outcomes (pain, discomfort, satisfaction).

Outcome assessors were not blind, but a dedicated blinded statistician interpreted the data, ensuring a blind process and eliminating bias. The study concluded on 31 December 2024.

## 3. Results

The registry defined a target of 100 participants (50 per arm). Sample size calculations were performed for each primary outcome scale using a two-sided two-sample *t*-test approximation with α = 0.05 and equal allocation as shown in Table 3. Cohen’s d was computed as the absolute difference between group means divided by the pooled standard deviation. Achieved power was estimated for the current design (*n* = 50 per group). For planning purposes, the required sample size per group to achieve 80% power was obtained for each scale. Although non-parametric tests (Mann–Whitney U) were applied in the present analysis due to non-normality, the *t*-test approximation was used here as a standard approach for sample size planning in future definitive trials.

A total of 100 patients were evaluated, divided equally into two groups: 50 patients in the intervention group (PRP Treated) and 50 in the control group (No treatment) as seen on Figure 1 and Table 1. Scar assessment was performed at two time points: day 7 and day 40 post-procedure using six validated scales as shown in Table 2 and Table 4.

For each scale, we compared the scores between the two groups using appropriate statistical tests based on the normality of data distribution. Normality was assessed using the Shapiro–Wilk test. For normally distributed variables, independent samples *t*-tests were used; otherwise, the Mann–Whitney U test was applied. For post hoc power and sample size calculations, *t*-test approximations were employed despite the use of non-parametric analyses in the main results. This approach was chosen because standard power calculations for non-parametric tests are less established, and *t*-test approximations provide a reasonable estimate for planning purposes in pilot studies.

Clinically, scars in the PRP-treated group appeared less erythematous and more uniform in color and texture at both 7 and 40 days postpartum. Edema and local induration were minimal compared with the control group, in which mild redness and localized thickening at the incision line were more frequently observed. By day 40, most PRP-treated scars demonstrated smoother contour and better pliability, consistent with early maturation signs, whereas control scars showed slightly elevated margins and residual hyperpigmentation in several cases.

On day 7, scores from the POSAS (both physician and patient assessments), Vancouver, Manchester, REEDA, NRS, and VAS were collected. Although the intervention group showed slightly lower means across most parameters (e.g., POSAS Physician: 8.88 vs. 9.28; Vancouver: 1.74 vs. 2.54) as shown in Table 4, none of these differences reached statistical significance (*p* > 0.05). The largest difference was observed for the Vancouver score (*p* = 0.064), indicating a non-significant difference with a possible direction of effect favoring the intervention group. This observation should be interpreted cautiously given the exploratory, underpowered design of this pilot study.

By day 40, scores generally decreased in both groups, reflecting scar maturation. The intervention group showed improved outcomes across most scales (e.g., POSAS Physician: 6.46 ± 1.23 vs. 6.84 ± 1.39), while the POSAS 40-day Patient score demonstrated a statistically significant difference favoring the PRP group (7.24 ± 1.81 vs. 8.00 ± 2.06; *p* = 0.029). Other comparisons did not reach statistical significance.

Effect sizes (Cohen’s d) was computed from group means and pooled SD for all outcomes. Across the board, effect sizes were small to moderate (<0.5), consistent with the observed *p*-values.

Among the outcomes, the largest standardized effects were observed for: Manchester 40D, POSAS 40D Patient, Vancouver 7D, and REEDA 7D. Detecting effects of this magnitude with 80% power would typically require between ~70 and ~140 participants per group as shown in Table 3.

## 4. Discussion

Platelet-rich plasma (PRP) is an innovative adjunctive therapy designed to accelerate tissue repair and reduce inflammation through the stimulation of growth factors that enhance regeneration and improve scar quality [9,11]. PRP has been shown to increase wound healing rates by approximately 20% and improve scar repair quality by 15–20% [12]. Previous studies have reported that PRP accelerates tissue recovery [13] and reduces postoperative complications in high-risk patients, including those with hypertrophic scar formation predisposition undergoing cesarean section [14]. Although similar pilot studies have investigated PRP in cesarean wound management, the present work extends these findings through a more comprehensive methodology. By combining intrauterine and subcutaneous PRP application with multiple validated scar assessment tools, it offers a more detailed evaluation of both clinical and patient-centered outcomes, thus contributing incremental but meaningful knowledge to the existing literature. It should be noted that the present protocol involved a single intraoperative PRP application. While this design ensured sterility, standardization, and minimal patient burden, evidence from other medical fields suggests that repeated PRP administrations may enhance tissue regeneration and scar remodeling. Future comparative studies should therefore explore whether multiple applications could achieve greater or more sustained benefits in cesarean wound healing.

The present study evaluated the effectiveness of PRP on cutaneous scar healing using a multimodal approach, incorporating objective clinical scales (Manchester, Vancouver, REEDA), patient-reported outcomes (POSAS, VAS, NRS), and physician-based assessments at two standardized postoperative time points.

Although differences between the intervention and control groups did not reach statistical significance, consistent trends favored the PRP group. Specifically, lower average scores were observed in the PRP group across most scales (POSAS, Vancouver, REEDA), reflecting improved scar appearance, reduced inflammation, and decreased discomfort. Among the evaluated scales, the POSAS 40-day Patient score reached statistical significance (*p* = 0.029), supporting a measurable improvement in patient-perceived scar quality following intraoperative PRP administration. All other scales showed favorable but non-significant trends.

At seven days postoperatively, the mean scar score was 8.88 ± 2.13; at 40 days, it decreased to 6.46 ± 1.23. The Wilcoxon test confirmed a significant improvement over time (*p* < 0.001). Scale-specific observations included:

Manchester scale: Marked reduction in edema and inflammation at 40 days, with improved scar thickness and appearance.

POSAS: Lower patient-reported discomfort at 40 days, suggesting effective tissue regeneration.

Vancouver scale: Notable improvement in elasticity and reduction in scar thickness at 40 days.

VAS and NRS: Significant pain reduction at 40 days; in other studies, PRP-treated patients showed a 93% VAS score reduction versus 79% in controls [13].

REEDA: Reduced inflammatory signs in all PRP-treated cases at 40 days, with an 85.5% reduction compared to 72% in controls [15].

These findings align with prior evidence indicating that PRP improves both clinical and hematological parameters, reflecting decreased inflammation and enhanced regeneration [16,17,18]. Mechanistically, PRP delivers platelet-derived growth factors that support all three phases of wound healing by stimulating fibroblast proliferation and collagen synthesis [19,20].

Recent systematic reviews and meta-analyses have further supported the potential role of PRP in wound healing.

For instance, Ebrahimi et al. (2022) concluded that PRP significantly improves scar quality and patient satisfaction across multiple surgical contexts, though results vary depending on PRP preparation and application timing [21]. Similarly, Hosseini et al. (2023) emphasized PRP’s regenerative potential through modulation of inflammatory and angiogenic pathways in post-burn and surgical wounds [11]. In obstetric surgery, Tehranian et al. demonstrated earlier wound epithelialization and reduced pain among PRP-treated cesarean patients, consistent with the favorable trends observed in our study [13]. However, as confirmed by the meta-analysis of Ebrahimi et al., most included trials are small-scale and heterogeneous, underscoring the need for standardized, adequately powered randomized studies such as the present one. Our results therefore contribute to this evolving evidence base by reinforcing the hypothesis that intraoperative PRP may offer clinical benefit in cesarean wound recovery, while also highlighting the methodological rigor needed for future multicenter validation.

From a mechanistic standpoint, PRP is known to contain a concentrated mixture of growth factors, including platelet-derived growth factor (PDGF), transforming growth factor-beta (TGF-β), vascular endothelial growth factor (VEGF), and epidermal growth factor (EGF), which are essential mediators of wound healing [9,19,20]. These bioactive molecules play pivotal roles in fibroblast proliferation, collagen synthesis, and angiogenesis while modulating the inflammatory response [21,22]. In the context of cesarean section, PRP may help shorten the inflammatory phase by reducing neutrophil infiltration and promoting an earlier transition to the proliferative phase, thereby accelerating tissue regeneration.

While statistical significance was not reached (*p* > 0.05), moderate effect sizes were observed—particularly for the Vancouver score at day 7 (Cohen’s d ≈ 0.5)—suggesting that the study may have been underpowered to detect subtle but clinically meaningful differences. Such underpowering is a common challenge in wound healing trials due to variability in patient-reported outcomes and biological response [23,24]. Furthermore, although some comparisons yielded *p*-values near the conventional threshold for significance (*p* = 0.063), these do not indicate true statistical significance. Instead, they suggest potential directions of effect that require validation in larger, adequately powered studies to avoid overinterpretation of exploratory findings.

From a methodological perspective, the combined use of multiple validated scar assessment tools enhances the robustness of these findings. POSAS uniquely integrates objective scar characteristics with patient-perceived outcomes, making it valuable in postpartum care [25]. Similarly, REEDA offers a practical and sensitive measure of early inflammatory changes, with good correlation to histological findings [26].

Given its low cost, autologous nature, and safety profile, PRP may represent a feasible adjunct to enhance postoperative scar management. However, its role in significantly improving broader clinical outcomes remains to be confirmed in larger, adequately powered multicenter trials.

It should be noted that scar remodeling is a prolonged biological process that continues for several months or even years after the initial injury. Therefore, our follow-up at 7 and 40 days primarily captures the early inflammatory and proliferative phases of wound healing rather than the long-term maturation and remodeling phase. Future studies with extended follow-up (e.g., 3, 6, and 12 months) are required to assess the durability of PRP’s effects on scar quality.

### 4.1. Clinical Implications

The findings of this pilot study, while not reaching statistical significance for most evaluated outcomes, suggest a consistent trend toward improved scar quality, reduced pain, and diminished inflammatory signs in patients receiving intraoperative PRP during cesarean delivery. From a clinical perspective, PRP may represent a simple, low-risk, and cost-effective adjunct to enhance postoperative recovery.

The potential benefits are particularly relevant for:

High-risk patients with delayed wound healing (e.g., obesity, diabetes, smoking, history of keloids or hypertrophic scars).

Settings with limited access to advanced wound care products, where autologous PRP can be prepared from the patient’s own blood during surgery at minimal additional cost.

Postpartum quality of life improvement, as better scar outcomes may reduce chronic pain, discomfort, and aesthetic concerns that can impact psychological well-being.

If further validated in adequately powered multicenter trials, PRP could be integrated into enhanced recovery after surgery (ERAS) protocols for cesarean deliveries, thereby standardizing its application and maximizing patient benefit.

### 4.2. Future Directions

To confirm and expand upon these preliminary observations, future research should:

Increase sample size–multicenter randomized controlled trials with at least 100 patients per group to ensure adequate statistical power for small-to-moderate effect sizes.

Implement full blinding–blinding both outcome assessors and patients to reduce potential detection and performance bias.

Include long-term follow-up–assessing scar quality, pain, and functional outcomes beyond the early postoperative period (e.g., 3, 6, and 12 months).

Conduct subgroup analyses–stratifying patients by risk factors such as BMI, parity, comorbidities, and type of skin closure to identify populations with the greatest benefit. Analyzing subgroups of high-risk patients—such as those with obesity, diabetes mellitus, or smoking habits—could provide more precise insights into which clinical profiles derive the most significant benefit from PRP-assisted cesarean healing.

Evaluate cost-effectiveness–integrating health-economic assessments to determine the financial feasibility of routine PRP use in obstetric care.

Explore biological mechanisms–incorporating histological, biochemical, or imaging-based endpoints to better understand PRP’s effects on collagen deposition, angiogenesis, and inflammation modulation.

The goal should be the development of standardized PRP preparation and application protocols tailored for obstetric surgery, accompanied by clear clinical guidelines and training modules for surgical teams. Future research should also assess the impact of treatment frequency, directly comparing single intraoperative PRP infiltration with serial postoperative applications to determine the optimal protocol for scar remodeling and patient recovery.

## 5. Conclusions

This single-center randomized controlled pilot study explored the potential benefits of intraoperative platelet-rich plasma (PRP) administration on postoperative cesarean section scar healing using multiple validated clinical and patient-reported scales. Although no statistically significant differences (*p* > 0.05)] were observed for most outcomes, consistent trends favoring the PRP group were consistently noted across several measures, with the POSAS 40D Patient score reaching statistical significance, these findings are preliminary and hypothesis-generating, suggesting a potential positive effect of PRP on scar quality, pain reduction, and inflammation control in the postpartum period. However, given the pilot nature and limited statistical power of this study, conclusions should be interpreted with caution until validated in larger, adequately powered multicenter trials. Given the limited sample size and the consequent low statistical power for some outcomes, the results should be interpreted with caution and considered hypothesis-generating. Larger, multicenter randomized trials with adequate sample sizes are warranted to confirm these preliminary observations and to better define the clinical role of PRP in cesarean wound management.

If confirmed in adequately powered trials, intraoperative PRP application could represent a simple, low-risk, and cost-effective adjunct to enhance postoperative recovery after cesarean delivery.

## 6. Limitations

This study has several limitations that merit consideration. First, although prospectively registered (NCT06978010), two minor deviations occurred: emergency cesarean sections were excluded after registration to maintain procedural uniformity; and hematological parameters, initially listed as exploratory outcomes, were recorded for safety but not statistically compared between groups. Both deviations are transparently reported here and justified as methodological refinements undertaken before data analysis.

Another limitation is that the study was conducted at a single center with a relatively small sample size, which may constrain the generalizability of the findings. The monocentric design limits external validity, particularly considering potential regional variations in obstetric practices and patient demographics.

Third limitation is the lack of blinding among outcome assessors introduces a potential risk of detection bias. Although data anonymization and blinded statistical analysis were implemented to reduce interpretive bias, these measures cannot fully substitute for a double-blind assessment protocol. Future investigations should therefore incorporate blinded or independent evaluators to enhance methodological rigor and minimize observer-related bias.

Fourth, potential confounding factors such as body mass index, smoking status, or socioeconomic determinants were not adjusted for in the analysis. Furthermore, the follow-up period was relatively short (limited to 40 days), capturing only the early inflammatory and proliferative phases of wound healing. Considering that full scar maturation may extend over several months or even years, the present results should not be extrapolated to long-term scar quality or durability.

In addition, no subgroup analyses were performed for specific high-risk categories (e.g., obesity, diabetes mellitus, or smoking), as the available sample was insufficient to allow reliable statistical stratification. These patient characteristics are known to influence wound healing dynamics and could reveal differential responses to PRP therapy that warrant exploration in larger, multicenter trials.

Moreover, this pilot trial was not statistically powered to detect small-to-moderate intergroup differences across the evaluated outcomes. Post hoc power estimations indicated that, for most scar assessment scales, the achieved power remained below the conventional 80% threshold (approximately 30–60% with the current sample size of 50 participants per arm). For instance, the POSAS 40-day patient score, which exhibited the largest between-group difference, yielded an effect size of *d* ≈ 0.39 and an estimated post hoc power of 49%. Sample-size projections suggest that at least 100 participants per arm would be required to detect such effects with 80% power at α = 0.05. Consequently, the current results should be interpreted as exploratory and hypothesis-generating, providing the basis for future adequately powered confirmatory studies.

Finally, the absence of photographic documentation represents an additional limitation. Although visual evidence could have strengthened the correlation between subjective and objective assessments of scar quality, its exclusion was motivated by ethical and privacy concerns, as abdominal surgical scars may be readily identifiable in postpartum patients. Subsequent research should aim to include standardized and anonymized photographic datasets to enhance the objectivity and reproducibility of visual outcome assessments.

## Figures and Tables

**Table 1:** Patients enrollment.

Group	Intervention Group (PRP Administration)	Control Group (No Treatment)
Number of Patients	50	50

**Table 2:** Outcome measures evaluated at 7 days (7D) and 40 days (40D) postpartum.

Variable
POSAS 7D Physician
POSAS 7D Patient
Vancouver 7D
Manchester 7D
REEDA 7D
NRS 7D
VAS 7D
POSAS 40D Physician
POSAS 40D Patient
Vancouver 40D
Manchester 40D
REEDA 40D
NRS 40D
VAS 40D

Note: For all scales, lower scores indicate better scar quality and less inflammation.

**Table 3:** Power and Sample Size Analysis.

Scale	Time Point	Mean (PRP)	SD (PRP)	Mean (Control)	SD (Control)	Cohen d	Achieved Power *n* = 50/Group	Required *n*/Group for 80% Power
POSAS 7D Physician	7D	8.88	2.13	9.28	2.72	0.164	0.128	587
POSAS 7D Patient	7D	10.08	3.28	11.0	4.0	0.252	0.238	250
Vancouver 7D	7D	1.74	1.58	2.54	2.3	0.405	0.519	97
Manchester 7D	7D	6.2	0.98	6.64	1.35	0.373	0.455	114
REEDA 7D	7D	0.88	1.09	1.56	2.1	0.406	0.521	96
NRS 7D	7D	1.98	1.43	2.38	1.53	0.27	0.267	217
VAS 7D	7D	1.08	0.89	1.3	1.32	0.195	0.162	412
POSAS 40D Physician	40D	6.46	1.23	6.84	1.39	0.29	0.3	189
POSAS 40D Patient	40D	7.24	1.81	8.0	2.06	0.392	0.492	104
Vancouver 40D	40D	0.46	0.73	0.76	1.18	0.306	0.328	169
Manchester 40D	40D	5.26	0.48	5.58	0.83	0.472	0.647	72
REEDA 40D	40D	0.18	0.43	0.24	0.51	0.127	0.097	972
NRS 40D	40D	0.1	0.36	0.6	0.8	0.806	0.979	26
VAS 40D	40D	0.04	0.19	0.14	0.4	0.319	0.353	155

**Table 4:** Used scales, mean score and standard deviation.

Variable	Intervention Group (Mean Score)	Intervention Group (Standard Deviation)	Control Group (Mean Score)	Control Group (Standard Deviation)
POSAS 7D Physician	8.88	2.13	9.28	2.72
POSAS 7D Patient	10.08	3.28	11.0	4.00
Vancouver 7D	1.74	1.58	2.54	2.30
Manchester 7D	6.2	0.98	6.64	1.35
REEDA 7D	0.88	1.09	1.56	2.10
NRS 7D	1.98	1.43	2.38	1.53
VAS 7D	1.08	0.89	1.3	1.32
POSAS 40D Physician	6.46	1.23	6.84	1.39
POSAS 40D Patient	7.24	1.81	8.0	2.06
Vancouver 40D	0.46	0.73	0.76	1.18
Manchester 40D	5.26	0.48	5.58	0.83
REEDA 40D	0.18	0.43	0.24	0.51
NRS 40D	0.1	0.36	0.6	0.80
VAS 40D	0.04	0.19	0.14	0.40

Note: For all assessment tools (POSAS, Vancouver, Manchester, REEDA, VAS, NRS), lower scores represent improved scar healing and reduced symptom severity.

## Data Availability

The authors confirm that the data supporting the findings of this study are available within the article.
